# Detection of SARS‐CoV‐2 in respiratory samples from cats in the UK associated with human‐to‐cat transmission

**DOI:** 10.1002/vetr.247

**Published:** 2021-04-22

**Authors:** Margaret J. Hosie, Ilaria Epifano, Vanessa Herder, Richard J. Orton, Andrew Stevenson, Natasha Johnson, Emma MacDonald, Dawn Dunbar, Michael McDonald, Fiona Howie, Bryn Tennant, Darcy Herrity, Ana Da Silva Filipe, Daniel G. Streicker, the COVID‐19 Genomics UK (COG‐UK) consortium, Brian J. Willett, Pablo R. Murcia, Ruth F. Jarrett, David L. Robertson, William Weir

**Affiliations:** ^1^ MRC‐University of Glasgow Centre for Virus Research Glasgow UK; ^2^ Veterinary Diagnostics Service, School of Veterinary Medicine University of Glasgow Glasgow UK; ^3^ SRUC Veterinary Services Pentlands Science Park Penicuik Midlothian UK; ^4^ Fareham Creek Veterinary Surgery Fareham Hampshire UK; ^5^ Animal Health and Comparative Medicine Institute of Biodiversity University of Glasgow Glasgow UK

**Keywords:** cats, COVID‐19, reverse zoonosis, SARS‐CoV‐2

## Abstract

**Objectives**: The aim of the study was to find evidence of SARS‐CoV‐2 infection in UK cats.

**Design**: Tissue samples were tested for SARS‐CoV‐2 antigen using immunofluorescence and for viral RNA by in situ hybridisation. A set of 387 oropharyngeal swabs that had been submitted for routine respiratory pathogen testing was tested for SARS‐CoV‐2 RNA using reverse transcriptase quantitative PCR.

**Results**: Lung tissue collected *post‐mortem* from cat 1 tested positive for both SARS‐CoV‐2 nucleocapsid antigen and RNA. SARS‐CoV‐2 RNA was detected in an oropharyngeal swab collected from cat 2 that presented with rhinitis and conjunctivitis. High throughput sequencing of the viral genome revealed five single nucleotide polymorphisms (SNPs) compared to the nearest UK human SARS‐CoV‐2 sequence, and this human virus contained eight SNPs compared to the original Wuhan‐Hu‐1 reference sequence. An analysis of the viral genome of cat 2 together with nine other feline‐derived SARS‐CoV‐2 sequences from around the world revealed no shared cat‐specific mutations.

**Conclusions**: These findings indicate that human‐to‐cat transmission of SARS‐CoV‐2 occurred during the COVID‐19 pandemic in the UK, with the infected cats developing mild or severe respiratory disease. Given the ability of the new coronavirus to infect different species, it will be important to monitor for human‐to‐cat, cat‐to‐cat and cat‐to‐human transmission.

## INTRODUCTION

SARS‐CoV‐2 belongs to the same species (*severe acute respiratory syndrome‐related coronavirus*) as the coronavirus responsible for the 2003 SARS epidemic. It emerged in December 2019, most likely from a bat reservoir in China, although a role for an intermediate species cannot be discounted.^1^, ^2^ During the current COVID‐19 pandemic, naturally occurring SARS‐CoV‐2 infections linked to transmission from humans have been reported in domestic cats,^3^, ^4^ nondomestic cats,^5^ dogs^6^ and mink.^7^ In addition, in vivo experiments have shown that while cats, ferrets and hamsters are susceptible to SARS‐CoV‐2 infection, ducks, chickens and pigs are apparently not susceptible.^8^, ^9^ Cat‐to‐cat transmission has been demonstrated experimentally,^8^, ^10^ but the significance of SARS‐CoV‐2 as a feline pathogen, as well as its reverse zoonotic potential, remains poorly understood. If SARS‐CoV‐2 were to establish new animal reservoirs, this could have implications for future emergence in humans.

At present, there is no evidence of cat‐to‐human transmission or that cats, dogs or other domestic animals play any appreciable role in the epidemiology of human infections with SARS‐CoV‐2.^11^ However, although the pandemic is currently driven by human‐to‐human transmission, it is important to address whether domestic animals are susceptible to disease or pose any risk to humans, particularly those individuals who are more vulnerable to severe disease. Cats often live very closely with their owners, licking their hands or faces, sometimes sleeping on or in their beds. There could also be risks associated with washing the cats' food and water bowls and cleaning out litter boxes. Domestic animals could also act as a viral reservoir, allowing continued transmission of the virus, even when R < 1 in the human population. Recent reports from Dutch mink farms of both mink‐to‐cat and mink‐to‐human transmission of the virus provide support for this scenario.^7^, ^12^ We used a range of laboratory techniques to show that two domestic cats from households with suspected cases of COVID‐19 and which displayed either mild or severe respiratory disease were infected with SARS‐CoV‐2. These findings confirm that human‐to‐cat transmission of SARS‐CoV‐2 occurs and can be associated with signs of respiratory disease in cats.

## MATERIALS AND METHODS

### Samples

Sections of lung tissue were collected *post‐mortem* from cat 1, placed in virus transport medium (VTM) and stored at –80°C on 22 April 2020; on 10 June 2020 the VTM was removed, and RNA*later* was added. Lung tissue was also stored in formalin from 22 April until 8 June, when it was processed to wax prior to immunohistochemistry.

Infection of cat 2 was identified via a retrospective survey of oropharyngeal and/or conjunctival swabs collected from 387 cats with respiratory signs that had been submitted to the University of Glasgow Veterinary Diagnostic Service (VDS) between March and July 2020 for routine pathogen testing.

### Ethics approval

Ethical approval for this study was granted by the University of Glasgow School of Veterinary Medicine ethics committee (EA27/20). Permission was given for the retrospective analysis of feline swabs submitted to VDS for routine respiratory pathogen testing. Permission was also granted for a public appeal to practising veterinary surgeons via the veterinary record, to solicit the submission of samples from suspect SARS‐CoV‐2 cases.^13^ This appeal was in line with guidance to veterinarians on the testing of animal samples for SARS‐CoV‐2 from the Animal and Plant Health Agency, issued on 13 May.^14^ This briefing note confirmed that testing of animals for the purpose of clinical research was permitted under appropriate ethical review.

On submitting samples to Scotland's Rural College (SRUC) Veterinary Services, veterinary practices agree that any sample may be used to investigate new and emerging diseases. Approval to test tissue samples collected *post‐mortem* from cat 1 in the study was obtained from the owner by the primary veterinary surgeon.

### Respiratory pathogen screening

A retrospective screening programme was undertaken, testing a set of 387 oropharyngeal swabs that had been submitted to the University of Glasgow VDS for routine testing for respiratory pathogens (feline calicivirus (FCV), feline herpes virus (FHV) and *Chlamydia felis* (*C. felis*). Samples received in VTM were screened for FHV, FCV and *C. felis*.

DNA extracts from VTM samples were tested for the presence of FHV and *C. felis* using a multiplex quantitative polymerase chain reaction (qPCR) approach. The assay incorporated published *C. felis* primers^15^ together with primers/probes for FHV and a feline host control gene that were designed in‐house. FCV isolation was attempted using feline embryonic cells, strain FEA,^16^ cultured in Dulbecco's modified eagle's medium (DMEM) supplemented with 10% foetal bovine serum, 2 mM L‐glutamine, 1 mM sodium pyruvate and 240 U mL^−1^ penicillin streptomycin (Gibco, Life Technologies, UK). Cells were maintained at 37°C in 5% CO_2_. Oropharyngeal swabs were taken into DMEM, and the medium was inoculated onto FEA cells; FCV isolation was recognised by the production of a characteristic cytopathic effect as described previously.^17^ The remnants of these samples were stored at 4°C prior to RNA extraction from each sample and testing for SARS‐CoV‐2 using RT‐qPCR.

### Immunofluorescence staining of tissue samples

Sections of 2–3 *μ*m thickness of formalin‐fixed and paraffin‐embedded (FFPE) lung and liver tissue were cut with a microtome and mounted on glass slides. After sodium‐citrate pressure cooking, the rabbit anti‐nucleocapsid antibody (NovusBio, code: NB100‐56683SS, dilution 1:100) and an AlexaFluor‐488 secondary antibody (ThermoFisher, code: A‐11034) as well as the ProLong Gold Antifade Mountant with DAPI (ThermoFisher, code: P36935) were used. For the detection of SARS CoV‐2 specific RNA encoding S by in situ hybridisation, the RNAscope 2.5 HD Reagent Kit‐RED (code: 322350, Advanced Cell Diagnostics) and the probe V‐nCoV2019‐S (code: 848561, Advanced Cell Diagnostics) were purchased and the protocol was followed according to the manufacturer's instructions. As positive controls (for immunofluorescence and *in situ* hybridisation), FFPE‐Vero cell pellets experimentally infected with SARS CoV‐2 were used and mock infected FFPE‐Vero cells served as negative controls.

### RNA extraction from respiratory samples and PCR amplification of SARS‐CoV‐2 genome

TRIzol Reagent (ThermoFisher Scientific, Paisley, UK) was added to lyse the sample and ensure inactivation of SARS‐CoV‐2, followed by organic solvent extraction using chloroform: isoamyl alcohol. Subsequent steps were performed using RNeasy Mini Kits (Qiagen, Manchester, UK) as per the manufacturer's instructions, with elution of the final RNA sample in 55 *μ*l nuclease‐free water. One mock RNA extraction was performed for every seven samples. All samples were tested using two reverse transcriptase‐qPCR (RT‐qPCR) assays: the 2019‐nCoV_N1 assay (https://www.fda.gov/media/134922/download) and an Orf1ab assay (primerset‐18, documented at https://tomeraltman.net/2020/03/03/technical‐problems‐COVID‐primers.html).

Primers and probe for the 2019‐nCoV_N1 assay were obtained ready‐mixed from IDT (Leuven, Belgium) and used at a final concentration of 500 nM and 127.5 nM, respectively. Primers and probe for the Orf1ab assay were synthesised by IDT and used at a final concentration of 800 nM and 400 nM, respectively. PCRs were performed in a final volume of 20 *μ*l including NEB Luna Universal Probe One‐Step Reaction Mix and Enzyme Mix (both New England Biolabs, Herts, UK) and 5 *μ*l of RNA sample. Thermal cycling was performed on an Applied Biosystems 7500 Fast PCR instrument running SDS software v2.3 (ThermoFisher Scientific) using the following conditions: 55°C for 10 min and 95°C for 1 min followed by 45 cycles of 95°C for 10 s and 58°C for 1 min. Four 10‐fold dilutions of SARS‐CoV‐2 RNA standards, which were quantified by comparison with plasmids containing the N1 sequences, were tested in duplicate with each PCR assay. Negative controls included the mock RNA extractions and at least two no‐template controls per 96‐well plate.

### Sequencing the feline SARS‐CoV‐2 genome

Following nucleic acid extraction, 5 *μ*l of the extract, corresponding to 81 genome copies, was utilised to prepare a library, following a protocol developed by the ARTIC network, adapted for Illumina sequencing. Briefly, the protocol described in https://www.protocols.io/view/ncov‐2019‐sequencing‐protocol‐v2‐bdp7i5rn was followed, until the amplicon generation stage, utilising the primer version 3. The resulting DNA amplicons were cleaned using AMPURE beads (Beckman Coulter) and libraries prepared using a DNA KAPA library kit (Roche) following the manufacturer's instructions. Indexing was carried out with NEBNext multiplex oligos (NEB), using seven cycles of PCR. Sequencing was performed in a MiSeq system using a MiSeqV2 cartridge (500 cycles), resulting in 93.57% of reads with Q score > 30. Reads were quality filtered with TrimGalore (https://github.com/FelixKrueger/TrimGalore), aligned to the Wuhan‐Hu‐1 reference strain (Gen‐Bank accession MN908947.3) using BWA^18^ followed by primer trimming and consensus calling with iVar.^19^ Negative controls processed in parallel retrieved no viral mapped reads after primer trimming. The created viral genome sequence for cat 2 was uploaded to GISAID with the accession number EPI_ISL_536400.

The closest UK human SARS‐CoV‐2 sequence was initially identified using the COG‐UK cluster identification tool civet (https://github.com/COG‐UK/civet). A maximum‐likelihood phylogenetic tree of all unique human SARS‐CoV‐2 sequences from the same county as cat 2 (*n* = 324), along with the cat 2 genome, the closest UK human sequence and the Wuhan‐Hu‐1 reference, was created using IQ‐TREE^20^ with the GTR substitution model (selected by IQ‐Tree ModelFinder) and 1000 bootstraps. Existing feline (*n* = 9; Belgium, China, France, Spain, USA) and mink (*n* = 13; Netherlands) SARS‐CoV‐2 genome sequences were downloaded from the GISAID website (https://www.gisaid.org) on 31 July 2020; a table of acknowledgements for these sequences is listed in supplementary materials.

## RESULTS

### Histopathological findings in cat 1 were consistent with viral pneumonia

**FIGURE 1:**
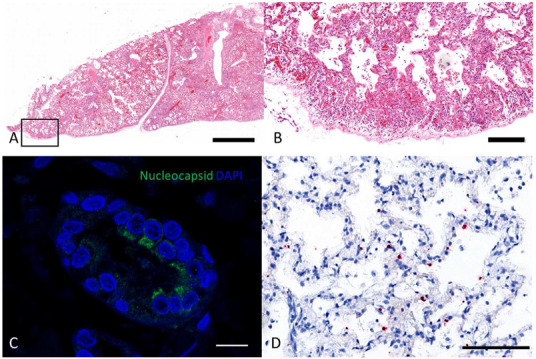
Section of lung of cat 1 stained with haematoxylin and eosin showing histopathological changes associated with viral pneumonia at low magnification (a; bar, 2 mm) with the area within the box shown at high magnification (b; bar, 200 *μ*m). A positive signal for nucleocapsid protein (green signal) was detected within the cytoplasm of the bronchiolar epithelium (c; bar, 10 *μ*m), and viral RNA (red dots) of the spike gene was detectable in alveolar membranes (d; bar, 100 *μ*m; haematoxylin counterstain)

Cat 1 was a 4‐month‐old female Ragdoll kitten from a household in which the owner developed symptoms that were consistent with SARS‐CoV‐2 infection at the end of March 2020, remaining symptomatic until 11 April 2020; the owner was not tested for SARS‐CoV‐2. The kitten was presented to its veterinary surgeon on 15 April 2020 with dyspnoea, and physical examination revealed signs of increased respiratory effort, increased respiratory rate and harsh lung sounds. Radiographic examination revealed an interstitial and alveolar pattern. The cat's condition deteriorated, and it was euthanised on 22 April 2020. Histopathological findings of the lung included a moderate to severe, diffuse, alveolar damage with sloughed pneumocytes, ruptured and thickened alveolar membranes, multifocal intra‐alveolar fibrin deposition, hyaline membranes, pneumocyte type II hyperplasia, and the presence of alveolar macrophages as well as thrombus formation within capillaries, arteries and veins. Mild to moderate amounts of inflammatory cells consisted of multifocally to diffusely distributed lymphocytes and macrophages. A few extravasated erythrocytes were present within the alveolar spaces, which were interpreted as mild haemorrhages, and moderate to severe congestion, as well as mild alveolar oedema was evident (Figure 1).

### SARS‐CoV‐2 antigen and RNA were demonstrated within pneumonic lung tissue

The presence of SARS CoV‐2 antigen was demonstrated following immunofluorescent staining of lung sections incubated with an antibody recognising the SARS‐CoV‐2 nucleocapsid (Figure 1). Antigen positive cells were detected in the bronchiolar epithelium, whereas no positive cells were detected in liver sections from the same cat. To rule out non‐specific immunofluorescent staining, in situ hybridisation was performed, using a probe targeting the viral spike gene. This demonstrated the presence of SARS‐CoV‐2 RNA in the lung; the positive signal was cell‐associated within the alveolar membranes, suggesting that type I pneumocytes were infected (Figure 1). In contrast, neither viral protein nor RNA was detected in the liver.

### Identification of SARS‐CoV‐2 RNA by retrospective surveillance of feline respiratory specimens

**FIGURE 2:**
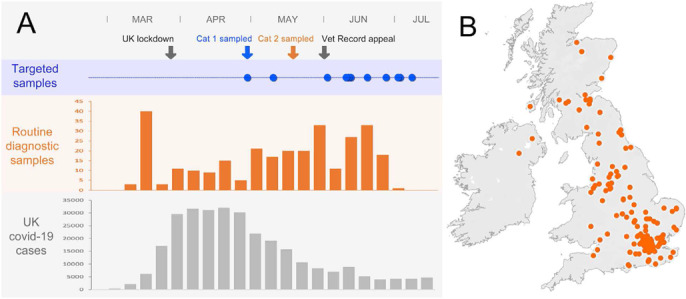
Timeline demonstrating the distribution of samples screened for SARS‐CoV‐2 and the testing strategy and sample collection relative to the UK pandemic

The sample collection for the retrospective screening of oropharyngeal swabs coincided with the period when community transmission of SARS‐CoV‐2 was widespread in the UK (Figure 2). Given the relatively low seroprevalence in humans (∼5%) at that time^21^ and, assuming that each cat sampled had an equal chance of coming from a COVID‐19 household, we estimate that approximately 19 of 387 samples in the collection came from cats belonging to COVID‐19 affected households.

Among 387 oropharyngeal swabs tested, the sample from one cat (subsequently designated as cat 2) tested positive using both the 2019‐nCoV‐N1 and the Orf1ab assays. All controls gave the expected results, and no other sample run on the same plate was positive (35 feline samples), ruling out potential laboratory contamination. The Ct values in the 2019‐nCoV‐N1 and Orf1ab assays were 34 and 33.5, respectively, representing a mean copy number of 81 viral genomes per 5 *μ*l of test sample. Any suggestion that cat 2 had been contaminated with SARS‐CoV‐2 from the owner was discounted as serum collected from the cat 8 weeks after the initial sampling tested seropositive in a live virus neutralisation assay conducted by an independent laboratory,^5^ confirming productive infection of cat 2.

The positive surveillance sample from cat 2 had been collected from a 6‐year‐old female Siamese cat that presented with bilateral yellow ocular discharge as well as a serous nasal discharge. Conjunctival and oropharyngeal swabs were collected from cat 2 on 15 May 2020 and sent to the University of Glasgow VDS to be tested for respiratory pathogens. The swabs tested positive for FHV DNA and negative by PCR for *C. felis*, and neither FHV nor FCV was isolated following attempted virus isolation on FEA cells.

One of the owners had symptoms consistent with COVID‐19 when cat 2 was presented to its veterinary surgeon with clinical signs. The cat tested positive for FHV DNA as well as SARS‐CoV‐2 RNA; the cat's clinical signs were consistent with FHV infection, and so the SARS‐CoV‐2 infection might not have been related to the clinical signs that the cat displayed at the time of sampling. However, it is also possible that co‐infection with SARS‐CoV‐2 caused reactivation of FHV in this cat.

### Comparison of feline and human SARS‐CoV‐2 genome sequences

**FIGURE 3:**
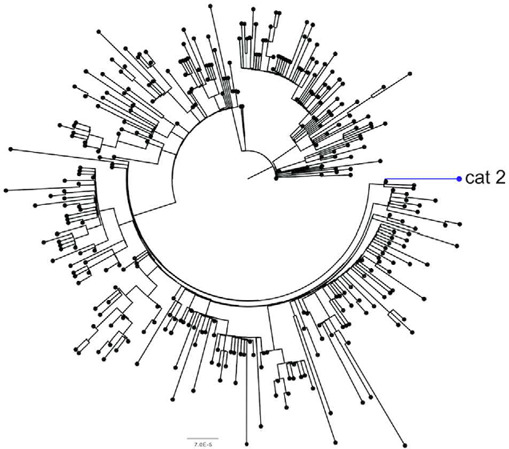
Maximum likelihood phylogenetic tree of the cat 2 SARS‐CoV‐2 viral genome (blue) with all unique human SARS‐CoV‐2 sequences from the same county as cat 2 (black). The tree is rooted on the Wuhan‐Hu‐1 reference sequence MN908947

To characterise cat 2's viral genome, we performed high‐throughput sequencing on RNA derived from the clinical specimen. The generated viral genome sequence was 97.2% complete and contained 13 single nucleotide polymorphisms (SNPs) when compared with the original Wuhan_Hu‐1 reference sequence. Sequence data from the symptomatic owner were not available; hence we compared the feline genome with other human SARS‐CoV‐2 sequences, using data from the COVID‐19 Genomics UK (COG‐UK) consortium. The mutational hamming distance (ignoring Ns and ambiguities) between the cat 2 viral genome and all COG‐UK human viral genomes available on 23 August 2020 revealed that the closest human SARS‐CoV‐2 sequences from the UK differed from the feline sequence by five SNPs (*n* = 141; Table 1); these human sequences were distributed throughout the UK but predominantly (88%)^22^ assigned to one lineage. The closest sequences (*n* = 11) from the same county as cat 2 were an additional SNP away. Phylogenetic analyses of these sequences reinforced the close relationship between the cat 2 viral genome and human‐derived UK SARS‐CoV‐2 genomes (Figure 3). As we do not have the owner's virus sequence, we cannot determine whether the observed mutations in cat 2's viral genome arose in a human prior to transmission.

**TABLE 1:** Details of the 13 SNPs observed in the cat 2 SARS‐CoV‐2 genome with respect to the original Wuhan‐Hu‐1 reference sequence, at least eight of which occurred in humans. Rows highlighted in bold represent the five SNPs not observed in the closest human sequences in the UK

Position	Gene	Mutation	AA Change	COG‐UK Human %	Feline %*	Mink %
241		C > T	non‐coding	17.88	0	53.85
3037	ORF1ab/nsp3	C > T	synonymous	82.22	100	53.85
3122	ORF1ab/nsp3	C > T	D135Y	0.01	0	0
14408	ORF1ab/nsp12	C > T	P323L	82.15	100	53.85
22330	S	A > C	synonymous	0	0	0
23403	S	A > G	D614G	82.37	100	53.85
25236	S	T > C/T	I1225T	0	0	0
25987	ORF3a	G > T	D199Y	0	0	0
27046	M	C > T	T175M	1.16	0	0
28312	N	C > T	synonymous	0.01	0	0
28881	N	G > A	R203K	52.88	40	0
28882	N	G > A	R203K	52.84	40	0
28883	N	G > C	G204R	52.84	40	0

* Three of the nine available feline sequences were less than 900 bases in length, and therefore feline percentages are calculated with respect to the sequences that cover each genome position.

Table 1 details the SNPs observed in the cat 2 viral genome and their frequency in the existing UK human population and among existing feline SARS‐CoV‐2 sequences. Six of the 13 SNPs are widespread (>50%) in the UK human population, and only three have not been observed previously. It is most likely that the three novel SNPs arose recently as evolutionary bottlenecks during human‐to‐human transmission and represent an unsampled cluster of human variants. Given that no other feline or mink sequences contained these mutations, there is little indication that these correspond to a host species adaptation of the virus.

Next, we examined all globally available feline SARS‐CoV‐2 sequences from the GISAID database for evidence of convergent mutations. Each of the six existing complete feline viral genomes contained three SNPs in common with cat 2 resulting in the D614G mutation in Spike, the P323L mutation in nsp12 and a synonymous mutation in nsp3. However, as these mutations are widespread in the human population, it is likely that they evolved in humans and are not associated with feline adaptation. The existing feline viral sequences were mutation distances of 0 (*n* = 4), 1 (*n* = 1), and 3 (*n* = 1) SNPs away from the closest human SARS‐CoV‐2 sequence in their respective countries. It has been suggested that the D614G mutation in spike (shared by the feline SARS‐CoV‐2 genomes) confers a fitness advantage to the virus in humans,^23^, ^24^ whether the same mutation renders the virus more infectious for cats remains to be established.

## DISCUSSION

This is the first report of human‐to‐cat transmission of SARS‐CoV‐2 in cats in the UK. Although the ongoing SARS‐CoV‐2 pandemic is driven by human‐to‐human transmission, concerns have been raised that other species might have the potential to play a role by becoming a new reservoir for the virus.^25^ Previously, there have been sporadic reports of human‐to‐pet transmission of SARS‐CoV‐2,^3^, ^4^, ^6^, ^26^ as well as human‐to‐wild felid^5^ and human‐to‐mink transmission.^7^ It is likely that such reports underestimate the true frequency of human‐to‐animal transmission since animal testing is limited and animals with subclinical infections are rarely, if ever, tested. Reverse zoonotic transmission represents a relatively low risk to animal or public health in areas where human‐to‐human transmission remains high. Nevertheless, as human‐to‐human transmission eventually wanes, prospects for transmission among animals become increasingly important as a source for re‐introductions to humans. It is therefore important to improve our understanding of whether exposed animals could play a role in transmission. An analysis of the feline SARS‐CoV‐2 genome from cat 2 demonstrated a high degree of sequence conservation with genomes derived from infected humans. We examined all of the reported feline SARS‐CoV‐2 sequence data and found no evidence of adaptation in the feline sequences. It is likely that all of the mutations in cat 2's viral genome were also present in the owner's virus, although the genome sequence of the owner's virus was not available for comparison. Whether SARS‐CoV‐2‐infected cats could naturally transmit the virus to other animals, or back to humans, remains unknown. Given the limited genetic variation observed to date among SARS‐CoV‐2 genomes from animals and humans and the evidence shown here that naturally infected cats shed virus (or at least moderate concentrations of viral RNA), it is highly likely that cat‐derived viruses could be transmitted to humans and to other animals. Recent outbreaks in Dutch mink farms provided further evidence of animal‐to‐animal transmission, and mink‐to‐human transmission has also been reported.^7^ Further studies are urgently required to determine the efficiency of animal‐to‐human transmission. It is recognised that, for ethical reasons and limitations on study design, it is unlikely that direct evidence of cat‐to‐human transmission could be obtained.^27^ It would not be feasible to conduct an experiment in which an uninfected person was exposed to an infected cat to determine whether cat‐to‐human transmission occurred. Therefore, it would be necessary to rely on evidence from the field; this would require that prior to exposure to an infected cat, a person would have observed an effective quarantine period, then they would have tested negative by PCR and serological testing (to eliminate the potential for undetected infection), and the person would have to remain isolated from all potential sources of SARS‐CoV‐2 other than the infected cat throughout the quarantine period. However, it will be important to investigate whether cat‐to‐human transmission is possible, or likely, and to determine the duration of virus shedding by infected cats and the level of contact with humans that is required for transmission to occur.

Cat 1 was euthanised because of the intractable progression of the dyspnoea in this kitten, whereas the milder clinical signs in cat 2 subsequently resolved. RNA was extracted from the lung of cat 1 and tested using only the 2019‐nCoV‐N1 RT‐qPCR assay to conserve the lung sample, since the amount of tissue was limited. The lung tissue tested positive, with the cycle threshold (Ct) value of 39 reflecting a low viral load. Illumina sequencing of the remaining RNA generated a consensus sequence representing only 30% of the genome, so sequence analyses were not performed. Nevertheless, the negative controls included in the sequencing run were negative, ruling out contamination and providing further confirmation of SARS‐CoV‐2 infection in the lung tissue of cat 1. Nevertheless, it is impossible to conclusively confirm that SARS‐CoV‐2 infection caused the viral pneumonia observed in this kitten, since the finding could have been incidental, and subclinical SARS‐CoV‐2 infections in cats from COVID‐19 households have been reported.^28^, ^29^, ^30^ Lung tissue was not tested for other potential causes of the pneumonia in this cat, e.g., FHV, FCV or Mycoplasma species; intranuclear inclusion bodies consistent with FHV infection were lacking.

Cat 2 tested positive for FHV and SARS‐CoV‐2; it is possible that coinfection with SARS‐CoV‐2 had led to reactivation of FHV in cat 2. Although no FHV was isolated from oropharyngeal and conjunctival swabs, FHV was detected by PCR, which is a more sensitive technique that, in contrast to virus isolation, is unaffected by sample degradation during transit.^31^ A second cat lived in the same household as cat 2, but neither SARS‐CoV‐2 RNA nor SARS‐CoV‐2‐specific antibody responses were detected in the second cat,^5^ indicating that the virus was not transmitted between these two animals that were living in the same household at the time of sampling. However, experimental transmission studies have shown that SARS‐CoV‐2 replicates efficiently in cats, causes severe disease in juvenile cats and can be transmitted from infected to sentinel cats via droplets.^8^, ^10^ The establishment of cat‐to‐cat transmission cycles could conceivably be suppressed or facilitated by local cat management practices, including the frequency of indoor/outdoor cats and the presence of feral cat colonies.

These two cases in the UK confirm previous findings that cats can acquire infection in households with SARS‐CoV‐2‐infected humans. One owner in the household to which cat 2 belonged tested positive for SARS‐CoV‐2, but no‐one from the household of cat 1 was tested. However, one person in the latter household had been symptomatic for approximately 2 weeks prior to cat 1 becoming dyspnoeic. Our findings highlight the importance of co‐ordinating the testing of humans and animals within affected households to monitor zoonotic transmission. The retrospective screening of 387 respiratory samples from cats led to the identification of a single cat that tested positive for SARS‐CoV‐2. However, it is unknown how many of these samples came from cats living in COVID‐19 affected households, and so we cannot estimate the frequency of human‐to‐cat transmission of SARS‐CoV‐2. Furthermore, the narrow window for detecting SARS‐CoV‐2 RNA decreases the likelihood of detecting zoonotic or reverse zoonotic virus transmission. Even though animal‐to‐animal transmission has been described ((8, 10), as well as a possible animal‐to‐human transmission,^12^ it is possible that animal‐to‐animal transmission could be greater under experimental conditions or in highly contaminated, crowded environments, such as mink farms. Variable rates of seropositivity among pet cats have been reported. Firstly, it was reported that 15 of 102 (14.7%) cat sera collected following the outbreak in Wuhan, China tested positive for antibodies that recognised the receptor binding domain of SARS‐CoV‐2 by ELISA, eleven of which (10.8%) also tested positive for neutralising antibodies.^32^ Next, a survey of pet cats and dogs from confirmed COVID‐19 households in France reported a high seroprevalence of SARS‐CoV‐2 antibodies, ranging from 21 to 53%, depending on the criteria used to define a positive result^33^ and in a longitudinal study of 76 cats living in COVID‐19 households in Texas, US, eight of 17 cats tested positive for SARS‐CoV‐2 RNA or neutralising antibodies (Hamer 2020). Also, in a study conducted in Northern Italy, it was reported that 5.8% of cats that were sampled at a time of frequent human infection were seropositive.^34^ In contrast, a German study suggested that human‐to‐cat transmission might be relatively infrequent; only 0.69% (6/920) of cats sampled between April and September 2020 for haematological testing were seropositive.^35^ Similarly, as few as 0.76% of cats presenting for routine veterinary visits in Croatia tested positive for neutralising antibodies.^36^ Furthermore, a study conducted in a veterinary community of 20 students, two of whom tested positive for COVID‐19 and eleven of the remaining 18 displayed symptoms of COVID‐19, demonstrated that none of the nine cats living in the community tested positive by RT‐PCR, and none of them developed antibodies.^37^ Therefore human‐to‐cat transmission appears to be highly variable, perhaps reflecting that transmission could be lower where good hygiene is practised. Hence further surveillance studies are needed to determine the prevalence of SARS‐CoV‐2 infection in cats, and whether future infections of cats represent spillover events from humans or are caused by sustained cat‐to‐cat transmission.

These findings have potential implications for the management of cats owned by people who develop SARS‐CoV‐2 infection. Currently, there is no evidence that domestic cats have played any role in the epidemiology of the COVID‐19 pandemic, but a better understanding of how efficiently virus is transmitted from humans to cats will require cats in COVID‐19 households to be monitored. The two cases of reverse zoonotic infections that are reported here serve to highlight the importance of a co‐ordinated one health approach between veterinary and public health organisations.

## ACKNOWLEDGEMENTS

This study was supported by an award to Margaret J. Hosie, Brian J. Willett, Ruth F. Jarrett, Pablo R. Murcia and William Weir from the Wellcome ISSF COVID Response Fund. Authors are supported by the Medical Research Council (MRC) of the United Kingdom: MC_UU_12014/9 (PRM), MC_UU_12014/9 (RJO and DLR), MC_UU_12018/12 (ADF). Ilaria Epifano is funded by the Chief Scientist Office (CSO) funding scheme, project code TCS/19/11. Daniel G. Streicker is funded by a Wellcome Trust Senior Research Fellowship (217221/Z/19/Z). Vanessa Herder is funded by the German Research Foundation (Deutsche Forschungs‐gemeinschaft), project number: 406109949. SRUC is supported by the Scottish Government. We gratefully acknowledge Lynn Oxford, Frazer Bell and Lynn Stevenson for technical assistance, the members of the COG‐UK consortium for sharing genome data and tools and all authors who have deposited and shared genome data on GISAID.

## AUTHOR CONTRIBUTIONS

Conceptualisation: Margaret J. Hosie, Pablo R. Murcia, Brian J. Willett, Daniel G. Streicker, Ruth F. Jarrett and William Weir. Methodology: Ilaria Epifano, Vanessa Herder and Ruth F. Jarrett. Formal analysis: Richard J. Orton, Ilaria Epifano and Ruth F. Jarrett. Investigation: Ilaria Epifano, Vanessa Herder, Andrew Stevenson, Natasha Johnson, Emma MacDonald, Dawn Dunbar, Michael McDonald, Fiona Howie and Bryn Tennant. Resources: Emma MacDonald, Dawn Dunbar, Michael McDonald, Darcy Herrity, Fiona Howie, Bryn Tennant and the COVID‐19 Genomics UK (COG‐UK) consortium. Writing‐original draft: Margaret J. Hosie, Vanessa Herder, Richard J. Orton, Ruth F. Jarrett, David L. Robertson and William Weir. Writing‐ review and editing: all authors. Visualisation: Ilaria Epifano, Vanessa Herder, Richard J. Orton, Fiona Howie and Bryn Tennant. Supervision: Margaret J. Hosie, Ana Da Silva Filipe, David L. Robertson, Ruth F. Jarrett and William Weir. Project administration: Margaret J. Hosie, Ana Da Silva Filipe, Ruth F. Jarrett and William Weir. Responsible for overall content: Margaret J. Hosie and William Weir.

**How to cite this article**: Hosie MJ, Epifano I, Herder V, Orton RJ, Stevenson A, Johnson N, et al. Detection of SARS‐CoV‐2 in respiratory samples from cats in the UK associated with human‐to‐cat transmission. *Vet Rec*. 2021;e247. https://doi.org/10.1002/vetr.247

## Supporting information

Additional supporting information may be found online in the Supporting Information section at the end of the article.

Supplement Material

Supplement Material
