# Supplementary material for: Detection of SARS‐CoV‐2 in respiratory samples from cats in the UK associated with human‐to‐cat transmission
Source: Vet Rec. 2021 Apr 22;188(8):e247. doi: 10.1002/vetr.247 (PMC8251078; doi:10.1002/vetr.247)
Supplement: Supplementary file 1 — Supplement Material [file VETR-188-no-s001.pdf]

We gratefully acknowledge the following Authors from the Originating laboratories responsible for obtaining the specimens, as well as the Submitting laboratories where the genome data were generated and shared via GISAID, on which this research is based.
Submitting laboratories where the genome data were generated and shared via GISAID, on which this research is based.
All Submitters of data may be contacted directly via www.gisaid.org

**Accession ID**	**Originating Laboratory**	**Submitting Laboratory**
EPI_ISL_420293	Wildlife Conservation Society, Bronx Zoo	Diagnostic Virology Laboratory, United States Department of Agriculture, National Veterinary Services Laboratories
EPI_ISL_421531	State Key Laboratory of Agricultural Microbiology	State Key Laboratory of Agricultural Microbiology
EPI_ISL_437349	Ecole nationale vétérinaire d'Alfort-laboratoire de santé animale Anses UMR 1161 de virologie ENVA-Anses-INRAE	Institut Pasteur CIBU-ERI
EPI_ISL_450406	Molecular Diagnostics, Antech Diagnostics	Molecular Diagnostics, Antech Diagnostics
EPI_ISL_450407	Molecular Diagnostics, Antech Diagnostics	Molecular Diagnostics, Antech Diagnostics
EPI_ISL_482820	Centre de Recerca en Sanitat Animal (IRTA-CReSA)	IrsiCaixa AIDS Research Lab
EPI_ISL_483063, EPI_ISL_483064	unknown	Virology, Ecole Nationale Veterinaire de Toulouse
EPI_ISL_487275	unknown	Department of Veterinary Pathology, University of Liege - FARAH
EPI_ISL_431778	Virology, Wageningen Bioveterinary Research	Virology, Wageningen Bioveterinary Research
EPI_ISL_447623, EPI_ISL_447624, EI	unknown	Virology
EPI_ISL_447631	unknown	Virology
EPI_ISL_447632, EPI_ISL_447633, EI	unknown	Virology
